# Dropping behaviour of pea aphid nymphs increases their development time and reduces their reproductive capacity as adults

**DOI:** 10.7717/peerj.2236

**Published:** 2016-07-27

**Authors:** Barbara Agabiti, Roxanne J. Wassenaar, Linton Winder

**Affiliations:** 1 Department of Natural Sciences, Unitec Institute of Technology, Auckland, New Zealand; 2 Department of Forestry and Resource Management, Waiariki Bay of Plenty Polytechnic, Rotorua, New Zealand

**Keywords:** Trade-off, *Acyrthosiphon pisum*, Dislodgement, Aphid, Non-consumptive, Population dynamics, Energy, Dropping

## Abstract

**Background.** Many aphid species, including the pea aphid *Acyrthosiphon pisum*, exhibit a behaviour where they drop or fall from their host plant, a commonly used strategy to avoid predation, parasitism or physical disturbance. We hypothesised that there was a physiological non-consumptive cost due to such dropping behaviour because aphids would expend energy re-establishing themselves on a host plant and also lose feeding time.

**Methods.** We evaluated this non-consumptive cost by determining the development time and reproductive potential of pea aphids that whilst developing as nymphs had regularly dropped to the ground following dislodgment from their host plant. Using a microcosm approach, in a replicated and balanced laboratory experiment, we caused aphid dropping behaviour by tapping the plants on which they were feeding.

**Results.** The results demonstrated that disturbance by dropping behaviour increased nymphal development time and reduced their subsequent reproductive capacity as adults.

**Discussion.** We conclude that dropping behaviour had a strong negative effect on the development of nymphs and their subsequent reproductive capacity. This implies that the physiological cost of such a behaviour choice is substantial, and that such avoidance strategies require a trade-off which reduces the capacity of a population to increase.

## Introduction

Many aphid species, including the pea aphid *Acyrthosiphon pisum* Harris 1776, exhibit a behaviour where they drop from their host plants as a response to disturbance (Gish, Dafni & Inbar, 2011), attack by natural enemies (Francke et al., 2008; Hoki, Losey & Ugine, 2014), or detection of alarm pheromone (Irwin, Kampmeier & Weisser, 2007). Such dropping behaviour can effect a substantial proportion of a population; in cereals, for example, dropping rates are typically in the range of 20–35% day^−1^ (Kerzicnik, Peairs & Harwood, 2010; Sopp, Sunderland & Coombes, 1987; Winder et al., 2013). The relative importance of disturbance caused by abiotic factors such as rainfall and abrasion (Jones, 1979; Ba-Angood & Stewart, 1980) compared to biotic factors is poorly understood, but both cause aphid dropping behaviour. Such dropping behaviour triggered by biotic cues primarily provides an escape mechanism from predation or parasitism. However, there must be an energetic cost associated with this behaviour, and therefore a consequent trade-off between escape from attack and the non-consumptive cost of an individual aphid re-establishing itself on a host plant by walking and climbing (Gish & Inbar, 2006; Nelson, Matthews & Rosenheim, 2004). The costs are likely to be twofold. Firstly, there is a risk that re-establishment does not occur at all due to predation by ground-active predators (Losey & Denno, 1998) or failure to re-climb due to desiccation and paralysis (Roitberg & Myers, 1979). Secondly, there is a physiological cost because exiting a feeding site reduces feeding time and energy is expended to find, re-climb a host plant and then recommence feeding (Phelan, Montgomery & Nault, 1976).

Nelson (2007) demonstrated that removing an aphid from a plant resulted in a physiological cost that reduced reproduction. In the experiment, pea aphids were separated from their host plant and then held in plastic vials temporarily (for 0, 1, 3 or 6 h) in a simulation of disturbance. This approach restricted aphid feeding but did not allow the aphids to expend energy to re-climb its host plant before beginning to feed. We hypothesised that a re-climbing cost is also incurred by such small and sedentary organisms, which would likely cause a reduction in aphid development rates. In this study, we evaluated this cost by determining the development time and reproductive potential of pea aphids that whilst developing as nymphs, had been deliberately dislodged from their host plant, causing them to exhibit dropping behaviour. Such studies are useful because they provide insights into the trade-offs that herbivores make between natural enemy avoidance and feeding (Thaler, McArt & Kaplan, 2012). Interactions between prey and natural enemies that influence fitness are key drivers that shape organismal traits (Langerhans, 2007). Such effects are driven by both consumptive (loss of individuals from a population) and non-consumptive (impact on survival, reproduction, and growth rates) natural enemy effects.

## Materials and Methods

*Acyrthosiphon pisum* were used as the model organism for this experiment, were reared in culture on broad bean (*Vicia faba* var. ‘Exhibition Long Pod’) seedlings in ventilated plastic cages (75 × 40 × 40 cm), and cultured in a controlled temperature growth room at 20 °C, 50–70% RH with a photoperiod of L16:D8 h. Lighting in the growth room was provided by a bank of horticultural grow-lamps (Grolite E40).

A main experiment was conducted in three phases: *Phase 1* (establishment of aphid colonies); *Phase 2* (nymphal development under varying levels of dropping behaviour); *Phase 3* (reproductive capacity as adults). We also conducted a subsidiary experiment in order to determine whether the method used to dislodge aphids causing the dropping behaviour (physically tapping the plants) confounded the main experiment by inadvertently inducing changes in host plant quality. Markovic et al. (2014) showed that brushing a plant (with a paintbrush) for 60 s a day altered plant chemistry sufficiently to cause aphids to express host plant avoidance. The subsidiary experiment was conducted in two phases, *Phase 1* (host plant conditioning) and *Phase 2* (reproductive capacity as adults).

### Main experiment

During *Phase 1*, thirty adult aphids were randomly selected from the stock culture and each established on a plant within a broad bean seedling microcosm. Aphids were carefully (individually) transferred to the microcosms using a small artist’s paintbrush. Each microcosm comprised a plant pot of 15 cm diameter containing potting compost and a clear plastic cylinder of 10 cm diameter and height 20 cm. The microcosm was sealed by pushing the cylinder into the soil surrounding the plant. The cylinder was wide enough to leave a space of c. 4–5 cm between the plant and the microcosm wall, was easily removable to allow access to the plant without disturbing the aphids, and was ventilated by provision of an opening covered by gauze at its top. Seedlings were at the four leaf stage and c. 4–6 cm in height. Each single adult aphid was left undisturbed to reproduce on the plant until ten nymphs were born. Each adult was then carefully removed from the microcosm.

During *Phase 2*, a replicated experiment was conducted where nymphs were subjected to three levels of disturbance (no, medium and high disturbance respectively) whilst developing to the adult stage. The thirty microcosms established in *Phase 1* were randomly allocated to the three levels of disturbance, with ten replicates for each treatment. Each microcosm was also randomly allocated a position within the experimental area of the growth chamber. The no disturbance (control) treatment nymphs remained undisturbed throughout *Phase 2*. Medium and high disturbance treatments were subjected to one (c. 9 AM) or two (c. 9 AM and 4 PM) dislodgement events that elicited dropping behaviour for five days each week. Dislodgement events were achieved by gently tapping leaves and/or stems (using a small artist’s brush) for sufficient time in order to elicit dropping behaviour (aphids fell to the ground). Each event was continued until >90% of the aphids had been dislodged, and it should be noted that aphids were not touched directly throughout. For the high dislodgement treatment, sufficient time elapsed between the two dropping events to ensure that aphids had returned to the plant prior to the second event. This experimental regime continued until adults emerged, at which point *Phase 3* was initiated. The number of days until adult emergence was recorded for each replicate and differences between development times investigated using one-way analysis of variance on untransformed data (which met the assumption of normality without transformation).

At the start of *Phase 3* (as soon as adults were observed), two newly emerged adults were transferred carefully to a new seedling in a new microcosm and left undisturbed to reproduce. The number of offspring were recorded daily until the end of the trial (i.e., when no further offspring were born). The total number of nymphs adult^−1^ produced was recorded and these count data were Log_10_(*n* + 1) transformed prior to analysis of variance to provide equality of variance. In addition, the intrinsic rate of population increase (*r*_*m*_, sensu Birch, 1948) was estimated using the equation derived by Wyatt & White (1977), where d (days) is the time taken for an adult to produce its first nymph, and *N* is the number of nymphs produced over the subsequent d days:

\begin{eqnarray*}{r}_{m}=0.74({\mathrm{log}}_{\mathrm{e}}N)/\mathrm{d}. \end{eqnarray*}

The mean intrinsic rate of increase (±1 s.e.), which represents the per capita rate of increase, was calculated for each treatment.

### Subsidiary experiment

*Phase 1* (plant conditioning) was initiated by establishing three host plant treatments (control, low tapping and high tapping). Each treatment was replicated five times on two trial occasions to yield ten replicates in total. Single plants were housed in microcosms identical to those used in the main experiment and were grown in the same growth room under similar conditions. Control treatments remained untouched, whilst the low and high treatments simulated the way plants were tapped in the main experiment. The low and high treatments were subjected to 30 s or two minutes of ‘tapping’ on one occasion daily (commencing at about 12 PM) respectively following the same methodology of the main experiment. This was repeated for five days, a duration comparable to that used by Markovic et al. (2014). *Phase 2* (aphid growth) commenced after the fifth tapping event. Colonies were established within each microcosm by carefully placing two fourth instar aphids (drawn from a stock culture) close to a plant stem. The colonies were then observed, and the number of adults established within each microcosm noted. *Phase 2* of the experiment continued for seven days and the number of nymphs born within each microcosm recorded and the mean number of nymphs produced adult^−1^ calculated for each microcosm. Count data were Log_10_(*n* + 1) transformed prior to analysis of variance. The ANOVA included both a treatment factor and a factor to represent the two experimental repeats.

**Figure 1:**
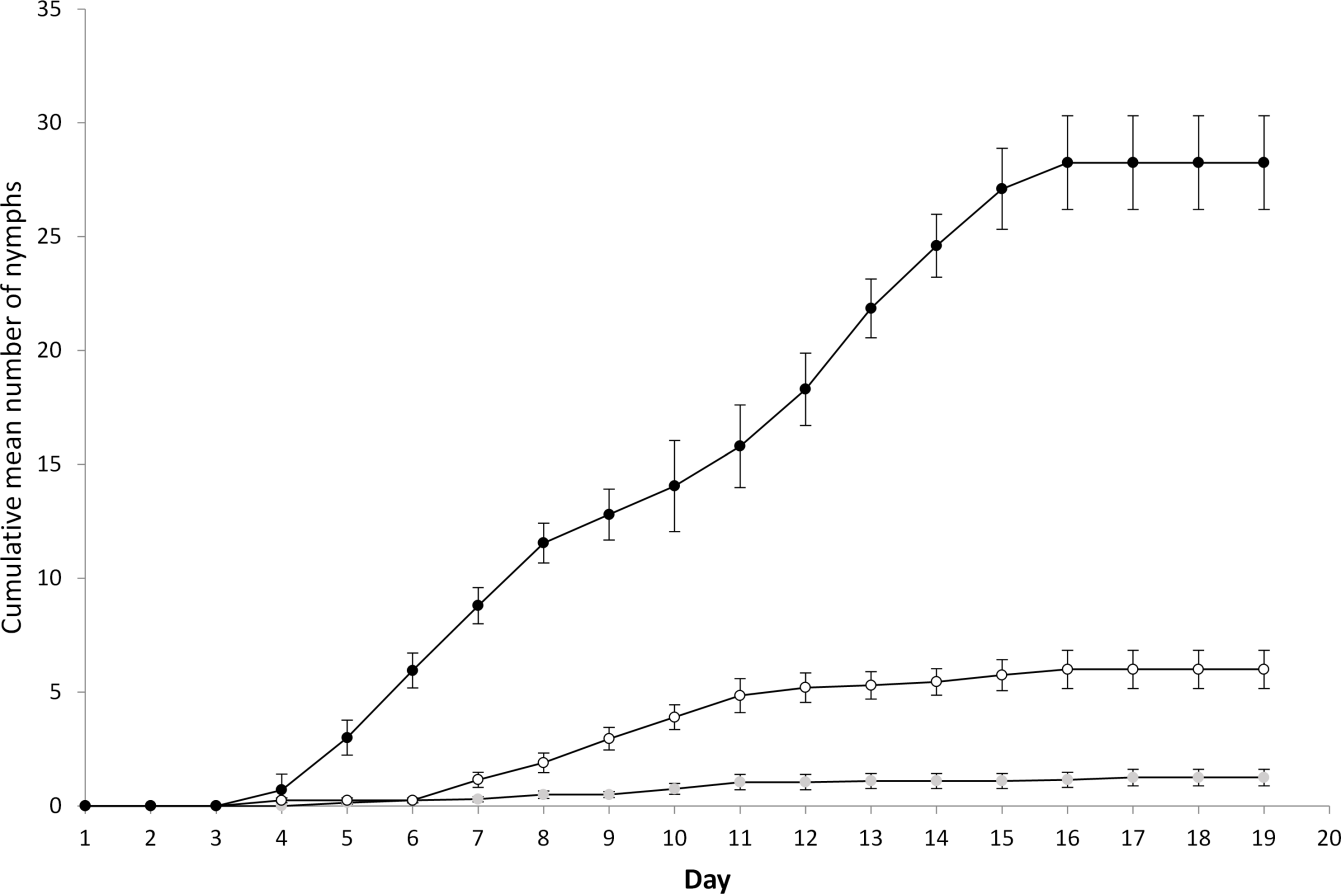
Cumulative mean number (±1 s.e.) of nymphs born adult^−1^ in no (filled black), medium (open black) and high (filled grey) disturbance treatments.

## Results and Discussion

For the main experiment, all replicates for each treatment were successfully initiated by establishing colonies of ten nymphs within each microcosm (*Phase 1*). During *Phase 2*, an average of 94% and 90% of aphids were displaced on each dropping event for the medium and high displacement treatments respectively. In order to achieve this level of displacement, plants were tapped repeatedly for two to five minutes. The mean number of days (±1 s.e.) before adulthood was 8.2 ± 0.4, 11.3 ± 0.4, and 11.3 ± 0.7 when subjected to no, medium and high disturbance respectively, demonstrating that disturbance resulted in a measurable increase in development time (*F* = 12.4, *P* < 0.001, d.f. = 2, 27). During *Phase 3*, the total mean number of nymphs produced adult^−1^ (±1 s.e.) subjected to no, medium and high disturbance was 28.3 ± 2.0, 6.0 ± 0.8, and 1.3 ± 0.4 respectively, demonstrating that with increasing disturbance there was a consequent measurable decline in reproductive capacity (*F* = 119.3, *P* < 0.001, d.f. = 2, 27). In all treatments the number of nymphs produced increased and then reached a plateau as the reproductive limit was reached (Fig. 1). No adult mortality was observed during this phase. Substantial differences in the intrinsic rate of increase was observed during the study. The intrinsic rate of increase (*r*_*m*_ ± 1 s.e.) was 0.56 ± 0.04, 0.05 ± 0.07, and −0.08 ± 0.03 when subjected to no, medium and high disturbance respectively. This result demonstrated that disturbance by dropping strongly reduced the capacity for population growth. It should be noted that the intrinsic rate for the high disturbance treatment was a slight overestimate as two replicates failed to produce any nymphs, and were therefore excluded from the analysis.

The supplementary experiment demonstrated that the method adopted to physically tap plants did not influence the subsequent reproductive success of aphids. In the supplementary experiment, no measurable difference in the number of nymphs born was evident between treatments (*F* = 0.81, n.s, d.f. = 2, 26) or between the two repeats (*F* = 0.35, n.s, d.f. = 1, 26) with the mean number of nymphs born being 35.4 ± 3.1, 33.8 ± 4.3 and 37.7 ± 3.9 for the control, low tapping and high tapping treatments respectively. This indicated that host plant quality was not affected by the experimental methods, although it is possible that aphids would have expressed host plant avoidance as observed by Markovic et al. (2014) had they been provided with a choice of host plant.

We conclude that the results demonstrated that dropping behaviour had a strong effect on the development of nymphs and their subsequent reproductive capacity. This implies that the physiological cost of such a behaviour choice is substantial, and that such avoidance strategies require a trade-off which reduces the capacity of a population to increase. Observations of the time taken for nymphs to return to the plant by re-climbing were typically 5–8 min at the start of *Phase 2* of the main experiment, a result broadly similar to aphid return times in other studies (e.g., Nelson, 2007 recorded a return within 1–2 min). Aphids were generally active and roamed prior to locating and then re-climbing the plant. Whilst the re-climbing time did not alter substantially for the medium disturbance treatment throughout the experiment, those observed in the high disturbance treatment took progressively longer, with some individuals taking as long as 1 h towards the end of the experiment. We conclude, therefore, that energy costs had the dominant effect (particularly at the start of the experiment), but, starvation costs increased in importance with repeated disturbance events. Return times were not a central focus of this experiment and further study of this phenomenon would be worthwhile, firstly to separate out the energy effects observed in this study and the starvation effects observed by Nelson (2007), and secondly, because, aphids may be exposed to further predation or other threats (i.e., desiccation) if they spend extended periods of time attempting to return to a host plant. Studies have shown that dropping behaviour leads to changes in spatial organisation (Minoretti & Weisser, 2000; Fievet et al., 2007; Winder et al., 2014) and such change is likely to influence the vulnerability of aphids to attack on host plants. Additionally, the tendency of different aphid instars to fall and the time taken for them to return to a host plant would provide further information with regards to the dynamics of non-consumptive effects.

Studies demonstrate that behavioural trade-offs are made by aphids. For example, Villagra, Ramiurez & Niemeyer (2002) showed that pea aphids changed their predominant anti-parasitoid response to *Aphidius ervi* from ‘walking away and dropping’ to a ‘kicking’ behaviour when subjected to different periods of food deprivation, indicating behavioural trade-offs mediated, in this case, by feeding stress. Similarly, Dill, Fraser & Roitberg (1990) showed that pea aphids were less likely to drop or walk in response to pheromones when feeding on high quality rather than on low quality hosts, and less likely to drop when the environment is hot and dry than when it is more benign. Additionally, studies demonstrate that aphid clones vary in their escape strategies (Braendle & Weisser, 2001; Schuett et al., 2015) indicating a range of life strategies amongst clones. These studies indicate complexity in the response of aphids to disturbance and the subsequent consequences for rates of population increase.

It could be fruitful to model population dynamics that integrate predation, parasitism and non-consumptive effects mediated by dropping behaviour. Non-consumptive behavioural effects (Fill, Long & Finke, 2012; Nelson, Matthews & Rosenheim, 2004) may have a profound effect on the population development of aphid (and other insect) populations and it may be that population models overestimate potential rates of increase if this factor is ignored. Nelson (2007) estimated that pea aphid birth rates could be reduced by 2–35% through such non-consumptive effects and our results suggest further reduction when repeated disturbance occurs. Models need to include parameters that account for changes in development and reproductive capacity due to such behaviours. This would help us understand more fully the benefits (or otherwise) of the behavioural trade-offs that aphids make when avoiding attack by natural enemies. Although our study is focussed on aphids, it is likely that other small insects with sedentary life histories may be affected in a similar way. Given that a substantial reduction in population growth characteristics were observed, it might be possible to develop pest control strategies that exploit such behaviour. Dropping behaviour elicited by mechanical disturbance or sub-lethal application of insecticides could potentially substantially reduce population development. Additionally, dropping behaviour would expose aphids to ground-active predators as well as abiotic challenges (such as wet soil) which could further suppress population development. Further study of the effects of disturbance on insects could provide additional insights into their population dynamics.

##  Supplemental Information

10.7717/peerj.2236/supp-1 Data S1 Raw data

## Additional Information and Declarations

### Data Availability

The following information was supplied regarding data availability: The raw data has been supplied as Data S1.
